# Loneliness in life and in death? Social and demographic patterns of unclaimed deaths

**DOI:** 10.1371/journal.pone.0238348

**Published:** 2020-09-16

**Authors:** Heeju Sohn, Stefan Timmermans, Pamela J. Prickett

**Affiliations:** 1 Department of Sociology, Emory University, Atlanta, Georgia, United States of America; 2 Department of Sociology, University of California, Los Angeles, Los Angeles, California, United States of America; 3 Department of Sociology, University of Amsterdam, Amsterdam, Netherlands; Indiana University Purdue University at Indianapolis, UNITED STATES

## Abstract

We examined family isolation, economic hardship, and long-distance migration as potential patterns of an extreme outcome of a lonely death: bodily remains that remain unclaimed and are left to the state. This paper combines a unique dataset—Los Angeles County's records of unclaimed deaths—with the Vital Statistics' Mortality data and the Annual Social and Economic Survey (ASEC) to examine 1) whose remains are more likely to become unclaimed after death and, 2) whether population-level differences and trends in family isolation, economic hardship, and long-distance migration explain the differences in the rates of unclaimed deaths. We employ multivariate Poisson models to estimate relative rates of unclaimed deaths by social and demographic characteristics. We find that increases in never married, divorced/separated, and living without family were positively associated with rates of unclaimed deaths. Unemployment among men and poverty among women was associated with higher unclaimed deaths. Long-distance migration was not associated with more unclaimed bodies.

## Introduction

Social relationships, particularly kin networks, significantly predict morbidity and mortality [1]. Individuals reporting no kin or social isolation from kin tend to suffer not only higher rates of physical and mental health but also increased mortality [2,3]. The presence of a spouse and regular contact with kin affects placement in nursing homes [4] and the dying experience [5]. We build on this research to examine a novel outcome of social isolation from kin: whether relatives will claim bodily remains. According to U.S. common law, the deceased have the right to a decent body disposition, and state laws specify that the right to possession of a dead human body remains with the relatives of the deceased, specifically the next-of-kin, with exceptions made for public welfare [6]. If the next of kin fails to act, state law designates government officials the right to dispose of the remains, at which point the deceased is considered "unclaimed". In Los Angeles County, where this study takes place, decedents are cremated and their unclaimed cremains placed in a common grave. Using a unique historical dataset, we investigate who is more likely to become unclaimed and whether demographic changes explain the differences in the rate of unclaimed over the past decades. Because the county where we conducted this study allows for a three-year period to reclaim cremains before they are permanently located in a common grave, we also investigate who is more likely to become reclaimed in that interim period.

In a country with strong normative preferences for body disposition by relatives [7], the rate of unclaimed bodies may reflect social isolation from kin. While the elderly compensate for a shrinking family network with alternative network ties [8,9], at time of death—as in life-threatening crises [10] and end-of-life decision making [11]—kin play a disproportionate decision-making role. Third parties, friends, and other non-kin, intending to claim a body face the high legal barrier of obtaining a court order. Thus, in this study of unclaimed deaths, we focus on isolation from kin rather than social isolation in a broader sense [12]. The only study published about unclaimed deaths in the U.S., using data from the Marion County coroner’s office 2004–2011, shows that all unclaimed had been identified, and in most cases, their next-of-kin had been notified of the death [13]. Indeed, except for jurisdictions that see extensive border deaths [14,15], state officials are able to locate relatives in the majority of unclaimed. For most unclaimed dead, the next-of-kin were unable or unwilling to take care of body disposition. Consequently, changes in the rate of unclaimed may reflect the extent to which close kin relationships have been stressed and severed at the time of death.

Shifts in the number of unclaimed deaths take place in a context of changes in family formation and dissolution [16–18]. The proportion of currently married adults has declined since the 1970s because of lower marriage rates and increased divorce, especially at older ages in recent decades [19]. Divorce and remarriage change the relationship between family members, weakening the support between parents and adult children [20]. Men are particularly vulnerable to a lack of kin support [21,22], especially after divorce [23,24]. Notably, adult children are less likely to support their aging fathers if they were divorced from their mothers [25]. These changes suggest a dwindling pool of family members that adults may rely on for support compared with same-age groups in prior cohorts. In terms of race, while some researchers posit that African Americans benefit from extensive kin networks [26], others have argued that socioeconomic factors have eroded social support among black families, especially for men [27].

Disposing of the dead is a financial imposition, and economic factors may also influence the rate of unclaimed deaths [28]. The median traditional adult burial cost $7,360 in 2017 [29]. Cremation is cheaper but not necessarily a bargain; the median cost was $6,260. We would, therefore, expect that the rate of unclaimed fluctuates with economic factors that affect both the deceased and the kin. If more deceased die without financial estates, they are less likely to have set aside the funds for a funeral. Financially strapped kin may not want to take on the unexpected expense of body disposition.

Dying far away from family may create additional barriers to claiming deaths. Claiming and transporting remains across the country increases the cost to burial. For foreign nationals, consulates often assist in claiming bodies, but the administrative and financial hurdles can be significantly higher than for domestic deaths. The proportion of the U.S. population that are immigrants increased from under 5 percent in 1970 to near 13 percent by 2010 [30]. Immigrants whose families also live in the U.S. may not face these additional barriers. However, a greater share of immigrants in the U.S. consists of recent immigrants due to (1) an accelerating rate of immigration into the U.S. since the 1960s [31], and (2) historically circulatory migration patterns driven by economic opportunities [32].

Fluctuations in the number of unclaimed bodies may thus reflect changes in family ties, changes in the economy, shifts in migration patterns. Our analysis tests the following hypotheses: (1) higher rates of unclaimed bodies are associated with disconnectedness from family (proportion never married, divorced/separated, and living without another family member), (2) rates of unclaimed bodies are higher among groups with greater unemployment and poverty, and (3) higher rates of unclaimed bodies are associated with greater domestic and international migration. We also tested differences across sex and race groups. Men often face harsher social consequences of unemployment and family dissolution [23,33], and the strain on the kin support network may be greater among race groups whose resources were already stretched thin [27]. We, therefore, examined sex- and race-interactions in the relationship between social trends and unclaimed bodies.

### Data and measures

We used the Los Angeles County Office of Decedent Affairs (LACODA) ’s unclaimed records with the Vital Statistics mortality data as the denominator to create an analytical dataset containing rates of unclaimed deaths by age, race, sex, and year. We then linked socioeconomic characteristics aggregated from the Annual Social and Economic Survey of the Current Population Survey (ASEC) to each demographic group and year. Los Angeles County is the most populous county in the United States and is larger than 41 U.S. States. As of 2018, the county’s population exceeded 10 million people and comprised 88 incorporated cities and unincorporated areas across 4,000 square miles. It is also one of the most ethnically and socioeconomically diverse counties in the United States [34].

LACODA has kept handwritten ledgers of the unclaimed remains’ sex, race, and age at death in addition to identifying information such as name and address since 1896. These handwritten entries are separate from death certificates and are not linked to individual Vital Statistics Mortality records. Unclaimed remains are cremated, and the cremains made available for pickup by kin for three years before being permanently buried in a common grave. LACODA notifies the next of kin of this policy with a letter to allow them an opportunity to claim the body even if they initially refused. We consider the deceased initially unclaimed and cremated by L.A. County but later retrieved by relatives within the three-year period as "reclaimed." About 17 percent of unclaimed remains were reclaimed and are noted in LACODA’s records. We took photos and digitized records from years ending in 3, 6, and 9 from 1973 to 2013. Gaining access to handwritten ledgers and digitizing them is an administrative- and labor-intensive process. Given the limited access and resources, we opted to select three years ending in 3, 6, and 9 from each decade to cover as wide a time frame as possible. LACODA unclaimed records contain non-veteran indigent deaths that were not claimed by surviving kin. Unclaimed veterans’ remains are handled by the Department of Veterans Affairs in conjunction with the LA County Medical Examiner-Coroner. As part of burial benefits, veterans are buried separately in national cemeteries and are not included in the county’s unclaimed records. The county’s Public Administrator handles cases where the unclaimed decedent’s estate is large enough to cover burial costs. Both groups make up a relatively small percentage of unclaimed deaths in L.A. County. Data gained under the California Public Records Act showed that out of 1,671 unclaimed deaths in 2014, 150 unclaimed veterans were transferred to Veterans Affairs, and 60 decedents were referred to the Public Administrator.

#### Rates of unclaimed deaths in L.A. County

We aggregated LACODA’s unclaimed deaths into combination groups by age, race, sex, and year. We then divided each cell by all deaths in L.A. County recorded in the Vital Statistics to derive rates of unclaimed bodies. We used the Center for Disease Control (CDC) ’s age group categorization. Deaths at age 85 and above were categorized as 85+. While LACODA handles all unclaimed remains, including stillbirths and newborns abandoned in medical settings, we limited our analyses to deaths of persons who were 15 or older. Race had three categories: white, African American, and other. Latin or Hispanic ethnicity was not recorded for deaths in the early years, and we did not differentiate ethnicity in our analyses. The way LACODA’s records categorized race was not standardized and changed over time. Many records noted the deceased’s presumed national origin (e.g., Vietnamese) rather than race per se. Some race categorization was inherently ambiguous (e.g., American). Thus, we decided to drop 332 records (1.82%) with ambiguous races. We followed the example of the early Vital Statistics and used white, African American, and other as our race categories and included deaths noted as Hispanic or Latino/a in the white category. We dropped 3.17 percent of unclaimed records that had missing or ambiguous demographic information. In sum, the analytical data of rates of unclaimed deaths have 648 total possible cells from a combination of two sex groups (male, female), three race groups (white, African American, and Other), nine age groups, and 12 years between 1976 and 2013. Nine of these cells (all are other race, female, and under 25) had no recorded deaths in the Vital Statistics. Both the LACODA and Vital Statistics data enumerate the complete population of deaths and are not subject to sampling errors. A subset of unclaimed bodies at LACODA was reclaimed by next of kin within the three-year grace period.

#### Social and demographic characteristics of L.A. County

We used the Annual Social and Economic Supplement (ASEC) from the CPS to derive key aggregate characteristics of Los Angeles County by age, sex, race, and year and linked them to rates of unclaimed deaths. We focused on three potential drivers of unclaimed deaths: isolation from family, economic hardship, and disconnection due to migration. We examined three measures of isolation from family: (1) proportion never married, (2) proportion divorced or separated, and (3) proportion who did not live with anyone related by blood or marriage. Each variable was aggregated and linked to each sex-race-age-year combination. Likewise, we derived proportions of unemployed (people who were not in the labor force are not considered unemployed) and living with incomes below the Federal Poverty Level (FPL) to examine the association between economic hardship and rates of unclaimed deaths. The third set of variables examines the link between long-distance migration and rates of unclaimed bodies. We used proportion moved to California within the past 12 months, proportion who immigrated to the United States, and proportion who were not U.S. citizens.

Our analytical data contained 614 cells containing rates of unclaimed deaths calculated from a full enumeration of 16,186 unclaimed remains and 683,907 deaths from Los Angeles County across 12 years between 1976 and 2013. The social and demographic characteristics were derived from the ASEC and aggregated and linked by sex, race, age, and year and linked to rates of unclaimed deaths by age, sex, race, and year. While the total possible number of cells is 648 (product of two sex, three race, nine age, and 12 year categories) 9 cells had no recorded deaths, and 25 cells had no survey respondents in the ASEC. Cells with no observations are concentrated among other race younger females (no recorded deaths) and among older non-White men (no ASEC respondents) (Table 1). Immigration-related variables were only available in the ASEC after 1995, and analyses using migration/immigration variables used 305 sex-race-age-year cells from 1996 to 2013.

**Table 1:** Descriptive characteristics of analytical data for Poisson models examining association between unclaimed bodies and socioeconomic characteristics.

		Number of cells in analytical dataset^1^	Number of total deaths^2^	Number of unclaimed bodies^3^	Percent of deaths unclaimed	Number of reclaimed bodies^4^	Percent of unclaimed bodies reclaimed
			(A)	(B)	(B/A)%	(C)	(C/B)%
**Total**		614	683,907	16,186	2.37	2,834	17.5
**Sex**	Female	307	331,134	5,106	1.54	976	19.1
	Male	307	352,773	11,080	3.14	1,858	16.8
**Race**	White	214	551,977	12,359	2.24	2,414	19.5
	African American	205	88,470	3,476	3.93	340	9.8
	Other	195	43,460	351	0.81	80	22.8
**Age**	15–19	66	6,203	45	0.73	4	8.9
	20–24	69	9,369	171	1.83	26	15.2
	25–34	72	22,167	716	3.23	144	20.1
	35–44	72	31,563	1,396	4.42	252	18.1
	45–54	72	53,854	2,527	4.69	500	19.8
	55–64	72	90,574	3,412	3.77	584	17.1
	65–74	72	136,499	3,529	2.59	598	16.9
	75–84	68	182,897	2,918	1.60	462	15.8
	85+	51	150,781	1,472	0.98	264	17.9
**Year**	1976	50	56,077	636	1.13	109	17.1
	1979	52	56,209	659	1.17	75	11.4
	1983	51	56,601	1,261	2.23	369	29.3
	1986	53	60,534	1,435	2.37	288	20.1
	1989	50	61,755	1,652	2.68	248	15.0
	1993	53	60,638	1,542	2.54	221	14.3
	1996	51	57,743	1,425	2.47	158	11.1
	1999	50	57,559	1,270	2.21	146	11.5
	2003	48	43,255	1,297	3.00	208	16.0
	2006	51	58,083	1,602	2.76	272	17.0
	2009	52	56,392	1,781	3.16	436	24.5
	2013	53	59,061	1,626	2.75	304	18.7

^1^ Number of observations in the analytical dataset for Poisson models. Total possible number of cells is 648 (all possible combinations of 2 sex, 9 age, 3 race, and 12 year categories). 9 cells had no deaths and 25 cells had no observations in the Annual Social and Economic Supplement (ASEC).

^2^ Source: Center for Disease Control (CDC) Mortality files.

^3^ Source: Los Angeles County Office of Decedent Affairs (LACODA) records of unclaimed remains. Data is available only for years ending in 3, 6, and 9.

^4^ Initially unclaimed bodies that were picked up by next-of-kin within the 3-year grace period are considered to be reclaimed bodies. Source: LACODA records of unclaimed remains

### Analytical strategy

Our primary analyses used Poisson regressions to estimate the relative rates of unclaimed deaths. We estimated the rate of unclaimed deaths by aggregating the number of unclaimed deaths from LACODA’s ledger into each sex-race-age-year group and matching it with total deaths in the equivalent group aggregated from the Vital Statistics mortality files. The Poisson models regresses the counts of unclaimed bodies in each group on social and demographic characteristics using total deaths as the exposure. Our first set of models (Models 1–4) estimates the relative rates of unclaimed bodies associated with demographic, family, and economic characteristics. Model 1 includes the basic demographic characteristics recorded by LACODA: sex, race, age, and year. Model 2 adds family and economic characteristics aggregated from ASEC to each sex-race-age-year group to Model 1. Model 3 adds sex interactions and Model 4 adds race interactions to the family and economic characteristics in Model 2. Models 1 through 4 uses data from years ending in 3, 6, and 9 between 1976 and 2013. LACODA data is only available for years ending in 3, 6, and 9, and ASEC begins in 1976. The ASEC includes immigration-related questions for years 1996 onwards. Models 5 through 7 that examine the relationship between long-distance migration and rates of unclaimed deaths are limited to years between 1996 and 2013. Model 5 estimates the relative rates of unclaimed deaths by groups’ migration histories while controlling for family and economic characteristics. Model 6 adds sex interactions, and Model 7 adds race interactions to the migration variables in Model 5. The last set of Models examines the rates of unclaimed bodies being reclaimed within the three-year grace period. The analytical data for Models 8 to 10 are limited to sex-race-age-year groups with at least one unclaimed body. Out of 614 cells between 1976 and 2013, 491 cells had at least one unclaimed body. Model 11 uses migration variables available from 1996 to 2013. 246 cells had at least one unclaimed body.

## Results

Table 1 enumerates total deaths, unclaimed bodies, and reclaimed bodies in Los Angeles County by key demographic characteristics. During the study period, unclaimed bodies accounted for about 2.37 percent of all deaths. 17.5 percent of unclaimed bodies were reclaimed by kin within three years. Percentages of deaths unclaimed by demographic characteristics show stark differences by sex, race, and age. 3.14 percent of male deaths went unclaimed compared to 1.54 percent of female deaths. African American deaths are unclaimed at higher rates (3.93 percent) than both white (2.24 percent) and other race groups (0.81). Percentages of deaths that went unclaimed are relatively low at the youngest and oldest age groups (0.73 and 0.98 respectively) but rise to 4.69 percent for deaths between 45 and 54. Rates of unclaimed bodies appear to rise over time. Percentages were around 1.15 percent in the late 1970s but rose to 3 percent in 2003 and peaked in 2009 at 3.16 percent. Surviving kin did not reclaim the majority of unclaimed bodies in our study period. Unclaimed female bodies were more likely to be reclaimed than male unclaimed bodies (19.1 percent versus 16.8 percent). Unclaimed African American bodies were the least likely to be reclaimed (9.8 percent) compared to white (19.5 percent) and other race groups (22.8 percent).

Table 2 provides descriptive summaries of key explanatory variables of Los Angeles County residents by sex, race, age, and year. Values are weighted by the number of deaths in each sex-race-age-year combination to reflect the characteristics of the underlying population of deaths in Los Angeles County. Characteristics, therefore, have greater representation from groups with a larger number of deaths (i.e., older groups). Overall, 10 percent of Los Angeles County residents were never married, and another 10 percent were divorced or separated. About 31 percent of residents did not live with a person related by blood or marriage, including siblings, children, parents, and other relatives. All three measures of isolation from family were greater among African Americans residents compared to whites and other race groups. Women and people in older age groups living in Los Angeles County were more likely to live without family compared to men and people in younger age groups. Percent unemployed was higher among men (3.1 percent), but percent living in poverty was higher among women (14.1 percent). Unemployment and poverty were the most prevalent among African Americans at 3.3 percent and 17.9 percent, respectively. Other races also had higher rates of poverty (14 percent) than whites (11.9 percent). Characteristics related to long-distance migration was only available for years 1996 onwards. While a significant proportion of Los Angeles County residents were immigrants (43.7 percent overall) less than 1 percent moved to California from out of state (including international) within the past year. Percent immigrant is particularly high (78.4 percent) among race groups that are not white or African American. About 40 percent of immigrants are not naturalized U.S. citizens. Rates are generally similar across sex and race.

**Table 2:** Descriptive socioeconomic, family, and migration characteristics in Los Angeles County, weighted by deaths^1^.

		1976–2013					1996–2013^2^		
		Family			Economic		Migration		
		Percent never married	Percent divorced or separated	Percent living apart from family	Percent unemployed^3^	Percent living in poverty	Percent moved from out of state in past 12 months	Percent immigrant	Percent non-US Citizen
**Overall**		10.0	10.1	31.2	2.0	12.8	0.6	43.7	18.1
**Sex**	Female	7.6	11.0	41.7	0.9	14.1	0.4	35.1	13.9
	Male	12.3	9.2	21.3	3.1	11.6	0.7	51.9	22.1
**Race**	White	9.2	9.5	32.3	1.9	11.9	0.5	45.2	19.0
	African American	16.5	15.3	32.5	3.3	17.9	0.6	11.1	5.5
	Other	6.9	6.2	14.4	1.7	14.0	1.1	78.4	28.6
**Age**	15–19	97.4	0.8	3.4	7.4	21.7	1.3	21.1	18.5
	20–24	80.4	2.5	20.1	9.0	16.6	3.0	34.1	28.8
	25–34	44.4	8.6	24.8	7.2	14.2	3.0	45.2	36.4
	35–44	20.2	15.1	17.5	6.4	14.3	1.4	46.0	30.9
	45–54	12.4	16.3	15.7	5.4	13.4	0.6	45.8	24.5
	55–64	9.2	14.9	19.4	3.4	11.9	0.4	44.2	20.5
	65–74	6.4	12.7	26.8	1.2	10.8	0.4	47.5	19.3
	75–84	3.9	8.4	35.9	0.4	11.3	0.5	42.2	14.9
	85+	5.1	4.6	47.5	0.5	15.6	0.5	42.2	12.7
**Year**	1976	7.4	9.1	35.1	2.3	12.0	na	na	na
	1979	11.3	9.4	30.9	1.4	9.4	na	na	na
	1983	7.1	8.5	29.2	2.7	12.1	na	na	na
	1986	11.0	10.2	31.1	1.2	10.2	na	na	na
	1989	10.8	10.4	35.6	1.4	11.5	na	na	na
	1993	12.1	7.6	27.0	2.5	15.4	na	na	na
	1996	11.0	9.6	32.8	1.9	15.8	1.4	42.2	26.2
	1999	8.2	10.2	27.7	2.2	14.2	0.2	43.4	19.7
	2003	11.8	13.5	28.0	2.1	11.7	0.9	48.1	21.4
	2006	9.5	12.7	33.0	1.3	13.2	0.2	42.3	15.5
	2009	9.3	9.6	30.0	2.3	10.3	0.6	41.5	15.1
	2013	10.8	10.6	32.7	3.4	17.4	0.4	45.8	11.3

Sample is limited to civilians aged 15 and above. Sample also excludes veterans.

^1^ Socioeconomic, family, and migration characteristics for each sex-age-race-year combination were aggregated from the Current Population Survey (CPS)'s Annual Social and Economic Supplements (ASEC). Values are weighted by number of deaths from the Vital Statistics and thus older age groups have greater representation in these values than in descriptive summaries that are weighted using population counts.

^2^ Immigration-related variables are only available in the CPS from 1996 onwards. Total possible number of cells is 324 (all possible combinations of 2 sex, 9 age, 3 race, and 6 year categories). Number of cells in analyses is 305; 12 categories had no observations in the ASEC and 7 had no deaths.

^3^ Unemployed does not include people who are not in the labor force.

Tables 3 and 4 contain the results of our main Poisson analysis. Table 3 examines the association between family isolation and economic hardship and rates of unclaimed deaths between 1976 and 2013. Model 1 first estimates relative rates of unclaimed deaths by sex, race, age, and year. Male deaths went unclaimed at a rate that is about 1.7 times that of female deaths. African American deaths were more likely to be unclaimed (1.415 times than whites), and controlling for other factors, deaths in the middle ages (35–54) were the most likely to be unclaimed. Model 2 estimates relative rates of unclaimed deaths associated with increasing family isolation and economic hardship while controlling for the demographic variables in Model 1. Living apart from family members is associated with greater rates of unclaimed bodies. A higher prevalence of singledom (never married and divorced/separated) is not significantly associated with rates of unclaimed deaths. While unemployment is significantly associated with greater rates of unclaimed bodies, poverty at the aggregate level is not. Model 3's sex interactions reveal significant sex differences in the association between family isolation and unclaimed deaths. Proportion never married is significantly associated with higher rates of unclaimed deaths among women but not men, and proportion divorced/separated is associated with higher rates of unclaimed deaths among men but not women. While the relationship between unemployment and unclaimed deaths were not significantly difference between men and women, living in poverty was associated with higher rates of unclaimed deaths for women. Model 4 explore race differences in the association between family isolation, economic hardship, and unclaimed deaths. The association between measures of family isolation and unclaimed deaths were not as strong among African American as they were among whites. Unemployment had no significant race interactions, but living in poverty had a significantly weaker link to unclaimed deaths among the ‘other’ race group.

**Table 3:** Poisson estimation of relative rates of bodies being unclaimed by family and economic characteristics 1976–2013.

*(relative ratios)*		Model 1	Model 2	Model 3	Model 4
**Family Characteristics**					
Proportion never married			1.13	1.87***	1.43*
Proportion divorced/separated			0.99	0.38***	1.55*
Proportion living away from family^1^			1.19*	1.07	1.23*
***Sex interactions (reference = female)***					
	Male x never married			0.57***	
	Male x divorced/separated			4.45***	
	Male x living away from family^1^			0.99	
***Race interactions (reference = white)***					
	African American x never married				0.73*
	African American x divorced/separated				0.57*
	African American x living away from family^1^				0.83
	Other x never married				1.84
	Other x divorced/separated				1.07
	Other x living away from family^1^				1.59
**Economic Characteristics**					
Proportion unemployed^2^			2.33***	1.11	2.94**
Proportion living in poverty^3^			1.13	1.62*	1.38
***Sex interactions (reference = female)***					
	Male x unemployed^2^			2.070	
	Male x living in poverty^3^			0.59*	
***Race interactions (reference = white)***					
	African American x unemployed^2^				0.69
	African American x living in poverty^3^				0.79
	Other x unemployed^2^				0.38
	Other x living in poverty^3^				0.18**
**Demographic Characteristics**					
Female		*(reference)*			
Male		1.71***	1.71***	1.55***	1.74***
White		*(reference)*			
African American		1.42***	1.36***	1.38***	1.69***
Other		0.30***	0.31***	0.31***	0.36***
15–19		*(reference)*			
20–24		2.53***	2.48***	2.59***	2.54***
25–34		4.47***	4.62***	4.77***	4.99***
35–44		6.12***	6.68***	6.82***	7.40***
45–54		6.75***	7.52***	7.74***	8.47***
55–64		5.70***	6.48***	6.70***	7.40***
65–74		4.06***	4.67***	4.76***	5.38***
75–84		2.59***	2.98***	3.06***	3.50***
85+		1.67***	1.87***	1.88***	2.21***
Year = 1976		*(reference)*			
Year = 1979		1.05	1.06	1.05	1.05
Year = 1983		2.04***	2.04***	2.06***	2.02***
Year = 1986		2.17***	2.21***	2.25***	2.18***
Year = 1989		2.46***	2.48***	2.50***	2.41***
Year = 1993		2.38***	2.36***	2.36***	2.30***
Year = 1996		2.37***	2.35***	2.34***	2.31***
Year = 1999		2.21***	2.21***	2.19***	2.14***
Year = 2003		2.48***	2.48***	2.47***	2.39***
Year = 2006		2.82***	2.80***	2.80***	2.73***
Year = 2009		3.27***	3.26***	3.25***	3.18***
Year = 2013		2.94***	2.84***	2.82***	2.71***
*Observations*		614	614	614	614
*Pseudo R-squared*		0.7	0.7	0.71	0.7

* p < .05,

** p < .01, and

*** p < .001

Poisson models use total deaths in each sex-race-age-year category as the exposure to unclaimed deaths. Family and economic characteristics are aggregated by each sex-race-age-year category. Relative ratios represent the multiplicative increase in the risk of being unclaimed associated with one unit increase in the covariate. For continuous family and economic covariates, one unit increase is equivalent to proportions increasing from 0 to 1. Analysis is limited to non-veteran civilians aged 15 and above. Data only available for years ending in 3, 6, and 9. Data sources: Annual Social and Economic Supplement (ASEC) of the Current Population Survey 1976–2013; Center for Disease Control (CDC) Mortality file 1976–2013; Los Angeles County Office of Decedent Affairs (LACODA) unclaimed bodies (1976–2013). Model 1 includes demographic variables only. Model 2 adds family and economic variables to Model 1. Model 3 adds sex interactions to Model 2, and Model 4 adds race interactions to Model 2.

^1^ Defined as people who are living in households without a person related by blood or marriage.

^2^ Does not include people who are not in the labor force.

^3^ Defined as people whose household incomes fall below the 100% Federal Poverty Level (FPL).

**Table 4:** Poisson estimation of relative rates of bodies being unclaimed by migration histories and immigration characteristics 1996–2013.

*(relative ratios)*		Model 5	Model 6	Model 7
**Migration/Immigration**				
Proportion moved to California within 12 months^1^		0.66	6.17	1.61
Proportion immigrant^2^		0.51***	0.78	0.43***
Proportion non-US Citizen^3^		2.15***	2.05*	2.62***
***Sex interactions (reference = female)***				
	Male x recently moved to CA		0.06	
	Male x immigrant		0.59*	
	Male x non-US Citizen		1.12	
***Race interactions (reference = white)***				
	African American x recently moved to CA			0.47
	African American x immigrant			1.63
	African American x non-US Citizen			0.94
	Other x recently moved to CA			0.020
	Other x immigrant			0.87
	Other x non-US Citizen			0.45
**Family and Economic Characteristics**				
Proportion never married		1.250	1.29	1.25
Proportion divorced/separated		0.85	0.93	0.81
Proportion living away from family^4^		1.09	1.08	1.07
Proportion unemployed^5^		2.66***	2.42**	2.69***
Proportion living in poverty^6^		1.33	1.32	1.35
**Demographic Characteristics**				
Female		*(reference)*		
Male		1.71***	2.05***	1.72***
White		*(reference)*		
African American		1.28***	1.30***	1.19*
Other		0.34***	0.33***	0.52
15–19		*(reference)*		
20–24		4.16***	4.20***	4.13***
25–34		10.79***	10.89***	10.81***
35–44		22.52***	22.76***	23.04***
45–54		29.22***	29.64***	30.22***
55–64		27.74***	28.20***	28.93***
65–74		20.38***	20.82***	21.62***
75–84		11.53***	11.96***	12.22***
85+		6.91***	7.34***	7.27***
Year = 1996		*(reference)*		
Year = 1999		0.97	0.98	0.99
Year = 2003		1.11*	1.10*	1.12**
Year = 2006		1.27***	1.26***	1.28***
Year = 2009		1.45***	1.45***	1.47***
Year = 2013		1.28***	1.28***	1.31***
*Observations*		305	305	305
*Pseudo R-squared*		0.75	0.75	0.75

* p < .05,

** p < .01, and

*** p < .001

Poisson models use total deaths in each sex-race-age-year category as the exposure to unclaimed deaths. Family and economic characteristics are aggregated by each sex-race-age-year category. Relative ratios represent the multiplicative increase in the risk of being unclaimed associated with one unit increase in the covariate. For continuous family and economic covariates, one unit increase is equivalent to proportions increasing from 0 to 1. Analysis is limited to non-veteran civilians aged 15 and above. Data only available for years ending in 3, 6, and 9. Immigration-related variables are only available for years 1996 onwards. Data sources: Annual Social and Economic Supplement (ASEC) of the Current Population Survey 1996–2013; Center for Disease Control (CDC) Mortality file 1996–2013; Los Angeles County Office of Decedent Affairs (LACODA) unclaimed bodies (1996–2013). Model 5 includes migration/immigration variables and controls for family, economic, and demographic characteristics. Model 6 adds sex interactions to Model 5, and Model 7 adds race interactions to Model 5.

^1^ Defined as people who lived in another state or country 12 months prior to survey date.

^2^ Defined as people who were not born as US citizens.

^3^ Does not include naturalized citizens.

^4^ Defined as people who are living in households without a person related by blood or marriage.

^5^ Does not include people who are not in the labor force.

^6^ Defined as people whose household incomes fall below the 100% Federal Poverty Level (FPL).

Table 4 examines migration and immigration characteristics in addition to the employment and family factors in Table 3. The models presented in Table 4 includes years from 1996 when immigration variables became available in the ASEC. The relationships between demographic characteristics—sex, race, age, and year—and unclaimed deaths in Models 5 to 7 in Table 4 show similar patterns to the Models in Table 3. However, the age pattern appears to be more pronounced in the recent period. Model 5 examines three migration-related variables—proportion recently moved from outside of California, proportion immigrant, and proportion U.S. citizen—in addition to the family isolation and economic hardship variables. While immigration overall is linked to lower levels of unclaimed deaths, the proportion of non-US citizens are linked to higher rates of unclaimed deaths. Model 6's sex interactions with migration variables show that the inverse relationship between immigration and unclaimed bodies is stronger among men than women. Model 7 shows no significant differences between race groups.

The final set of results (Table 5) estimates the relative rates of being reclaimed by kin within three years. Model 8 shows that male remains have significantly lower reclaimed rates than female remains (0.855), and African American remains are about half as likely to be reclaimed than white remains. Models 9 to 11 show a limited association between family isolation, economic hardship, and long-distance migration to rates of unclaimed bodies being reclaimed.

**Table 5:** Poisson estimation of relative rates of being reclaimed by socioeconomic characteristics.

*(relative ratios)*	1976–2013			1996–2013
	Model 8	Model 9	Model 10	Model 11
**Family Characteristics**				
Proportion never married		1.11	1.13	1.11
Proportion divorced/separated		1.16	1.17	0.88
Proportion living away from family^1^		1.17	1.170	1.98*
**Economic Characteristics**				
Proportion unemployed^2^			0.6	0.97
Proportion living in poverty^3^			1.01	0.46
**Migration/Immigration**				
Proportion moved to California within 12 months^4^				7.5
Proportion immigrant^5^				1.98
Proportion non-US Citizen^6^				1.64
**Demographic Characteristics**				
Female	*(reference)*			
Male	0.86***	0.87**	0.88**	0.78***
White	*(reference)*			
African American	0.50***	0.49***	0.49***	0.72*
Other	1.15	1.18	1.18	0.91
15–19	*(reference)*			
20–24	1.79	1.77	1.77	0.48
25–34	2.44	2.47	2.49	0.54
35–44	2.27	2.35	2.38	0.41
45–54	2.38	2.49	2.51	0.48
55–64	1.99	2.09	2.08	0.4
65–74	1.92	2	1.97	0.34
75–84	1.73	1.8	1.77	0.33
85+	1.87	1.91	1.88	0.29
Year = 1976	*(reference)*			na
Year = 1979	0.68**	0.67**	0.67**	na
Year = 1983	1.69***	1.69***	1.70***	na
Year = 1986	1.18	1.18	1.17	na
Year = 1989	0.87	0.86	0.85	na
Year = 1993	0.85	0.85	0.85	na
Year = 1996	0.66***	0.65***	0.65***	*(reference)*
Year = 1999	0.69**	0.69**	0.69**	1.13
Year = 2003	0.97	0.96	0.96	1.44**
Year = 2006	1	0.99	0.98	1.62***
Year = 2009	1.46***	1.46***	1.46***	2.39***
Year = 2013	1.13	1.11	1.12	1.92***
*Observations*	491	491	491	246
*Pseudo R-squared*	0.22	0.22	0.22	0.23

* p < .05,

** p < .01, and

*** p < .001

Poisson models use total unclaimed bodies in each sex-race-age-year category as the exposure to reclaimed bodies. Family and economic characteristics are aggregated by each sex-race-age-year category. Relative ratios represent the multiplicative increase in the risk of being unclaimed associated with one unit increase in the covariate. For continuous family and economic covariates, one unit increase is equivalent to proportions increasing from 0 to 1. Analysis is limited to categories with at least one unclaimed body and to non-veteran civilians aged 15 and above. Data only available for years ending in 3, 6, and 9. Immigration-related variables are only available for years 1996 onwards. Data sources: Annual Social and Economic Supplement (ASEC) of the Current Population Survey 1976–2013; Center for Disease Control (CDC) Mortality file 1976–2013; Los Angeles County Office of Decedent Affairs (LACODA) unclaimed bodies (1976–2013). Model 8 includes only demographic characteristics. Model 9 adds family characteristics to Model 8. Model 10 adds economic characteristics to Model 9. Model 11 adds migration/immigration variables to Model 10 and is limited to years 1996–2013; immigration variables were recorded in the ASEC after 1995.

^1^ Defined as people who are living in households without a person related by blood or marriage.

^2^ Does not include people who are not in the labor force.

^3^ Defined as people whose household incomes fall below the 100% Federal Poverty Level (FPL).

^4^ Defined as people who lived in another state or country 12 months prior to survey date.

^5^ Defined as people who were not born as US citizens.

^6^ Does not include naturalized citizens.

## Discussion

Rates of unclaimed deaths in Los Angeles County were less than 1.2 percent in the 1970s. Since the early 2000s, rates have fluctuated between 2.75 and 3.16 percent (Table 1). Our analyses revealed significant associations between family isolation, economic hardship, and rates of unclaimed bodies. Greater prevalences of living without a family member, being never married, and living divorced or separated were associated with higher rates of unclaimed deaths. In addition to our aggregate measures of family isolation, we also explored whether the decline in fertility may explain the rise in unclaimed bodies. We used the June Fertility Supplement to examine the trend in childlessness of women aged 15 to 44 who were living in Los Angeles County between 1994 and 2018. The CPS June Fertility Supplement is available for 1994, 1998, and every even year until 2018. Fertility histories (number of children even born) were only asked of women between ages 15 and 44. Due to the incompleteness of fertility data across sex, age, and year groups, we decided to not to include it in our primary analyses. The proportion of childless women under age 45 increased overall from about 42 percent in 1994 to about 58 percent in 2018. However, this trend likely reflects the delaying of age at first birth rather than reduced fertility; childlessness of 40 to 44-year-old women fell during the 20-year period between 1998 and 2018 from about 23 percent to 12.5 percent. The average number of children ever born, as well as childlessness, was the lowest among women born in the 1960s, yet rates of unclaimed deaths were higher among birth cohorts born in prior decades. The association (or the absence of one) between fertility and unclaimed deaths could not be conclusively explored however, due to the lack of complete data from both men and women across all age groups.

Periods of economic hardship may not only affect the deceased from arranging for their own burial plans but may also affect their surviving family members to pay for the costs of body retrieval and burial. We conducted supplementary analyses using annual unemployment rates for the entire county as a proxy for surviving kin’s economic status. High county-level unemployment was also strongly correlated with high rates of unclaimed bodies.

Contrary to our expectations, our analyses did not find a significant association between long-distance migration and unclaimed bodies. A greater prevalence of recent migrants to California from a different state or country was not associated with significantly higher rates of unclaimed bodies. Aggregate levels of immigration, in general, was associated with lower rates of unclaimed deaths, but the prevalence of immigrants who were not naturalized U.S. citizens was linked to higher rates of unclaimed bodies. A strong Catholic death culture among Latina/o immigrants [35,36]—the largest immigrant group—coupled with their likelihoods to reside in extended family networks upon arrival in the U.S. may provide protective factors against being unclaimed [30,37]. Naturalized immigrants, on average, have lived longer in the United States than non-citizens [38] and may likely have stronger familial ties in their adopted country.

We also examined domestic migration trends using available ASEC’s 5-year migration history variable in available years: 1980, 1985, 1995, 2005, and 2015. We were unable to merge these variables to LACODA’s unclaimed data as they only contained years ending in 3, 6, and 9. The proportion of respondents who lived in the same house steadily increased from about 44 percent in 1980 to 70 percent in 2015, and most movers moved within Los Angeles County. The decline in geographic mobility in Los Angeles is reflective of the national trend in internal migration since the 1980s [39] and moved counter to the increase in unclaimed bodies during the same period.

Demographic characteristics of deaths were also strong predictors of being unclaimed. Rates of unclaimed deaths were consistently higher for men than women and higher for African American than white deaths. Deaths between ages 45 and 54 have the highest rates of unclaimed deaths. The three-year reclaim rates were also significantly lower among men and African Americans compared to women and whites, respectively. Family, economic, and migration factors did not account for the large differences in unclaimed deaths between demographic groups.

Analyses with sex interactions with family and economic variables revealed notable sex differences; prevalence of divorce and separation predicted greater unclaimed deaths among men, and prevalence of the never married predicted greater unclaimed deaths among women. The link between unemployment and unclaimed bodies was stronger among men, while the link between poverty and unclaimed bodies was stronger among women. Race interactions show that the relationship between family isolation and unclaimed bodies was greater among whites than African Americans. Together, these findings reinforce prior literature on the social vulnerability of men, especially white men’s, kin ties after divorce [23,24].

These findings must be interpreted while considering the limitations of the data. First, the demographic characteristics of the unclaimed deaths are determined by the medical examiner-coroner and handwritten into a ledger. The way the records categorized people were not standardized, and they changed over time to reflect the language and culture of the period. Notably, deaths in the “other” race category increased throughout the study period. The increase may have been driven by the rising Asian population in Los Angeles County since the 1980s as well as the medical examiner-coroner’s preference for using more detailed ethnic categories (i.e., Native American, Samoan) than simply “Black” or “white”. Thus the generalizability and the interpretation of unclaimed bodies in the “other” race group is limited. Second, LACODA’s unclaimed data, as well as the Vital Statistics Mortality, do not contain information on the socioeconomic circumstances prior to the death. Therefore, we resorted to using aggregate measures from the living L.A. population taken from the ASEC as proxies. Thus, we cannot conclusively state that people with certain socioeconomic characteristics are more likely to be unclaimed. Rather, we observe compelling patterns that link the prevalence of family isolation and economic hardship in a particular age-sex-race-year group to the rate of unclaimed deaths in the same group. Third, the LACODA records of unclaimed deaths do not perfectly match the death certificates reported to the Vital Statistics. Thus, while both LACODA and the Vital Statistics each provide enumerations of unclaimed deaths, we expect a degree of error stemming from differences in categorization and record-keeping. LACODA records all unclaimed deaths that occurred in Los Angeles County (regardless of the residence of the deceased), whereas the Vital Statistics mortality database provides deaths by county of residence. Also, LACODA’s records of the exact date of death may differ from the Vital Statistics if bodies are discovered in non-medical settings (i.e., at home). Bodies found at the beginning of the year may be assigned to different years based on estimates of time of death. Fourth, the associations between unclaimed deaths and social, demographic, and economic characteristics that we observed in Los Angeles County may not generalize to the broader population of the United States. People from Mexico make up a large proportion of the immigration population in Los Angeles. The group’s strong norms for burial may not generalize to other immigrant groups in different parts of the United States. Los Angeles also has a larger than average population of Asian Americans who were categorized as "other" race in our analyses. About 15 percent of Angelenos in 2019 self-reported as Asian compared to about 6 percent nationally [34]. Rates of unclaimed deaths among the "other" race group will likely differ based on who is categorized in that group. Records of unclaimed bodies are often kept at the local level and while it would have been illuminating to examine trends at the national level, no such data exists. LACODA’s record of unclaimed bodies offers a rare opportunity to observe population-representative disparities in the very last stage of life.

Unclaimed deaths are extreme outcomes of social isolation and destitution at the end-of-life. The social relevance of the unclaimed reflects the connection between the deceased and their relatives. While we examined the outcome of remains after death, it likely captures the familial and economic circumstances in the years and months leading up to death. For the deceased, becoming unclaimed means that kin ties or economic resources were insufficient to organize a disposition. For relatives, allowing a kin member to go unclaimed reflects a shift against social expectations. While living kin claimed more than 95 percent of deaths in Los Angeles County, increasing non-marriage, divorce, and remarriage are changing the network and the nature of surviving kin at the end of life [40]. When facing financial scarcity, family members may be less likely to claim the bodies of estranged kin or kin indirectly related by marriage (i.e., stepfamily). The confluence of unstable family relationships and financial hardship among groups with already few socioeconomic resources makes them especially vulnerable to lonely deaths.
